# Temperature-dependent compatibility study on halide solid-state electrolytes in solid-state batteries

**DOI:** 10.3389/fchem.2022.952875

**Published:** 2022-08-03

**Authors:** Gaoshuai Jia, Zhi Deng, Dixing Ni, Zhaoran Ji, Diancheng Chen, Xinxin Zhang, Tao Wang, Shuai Li, Yusheng Zhao

**Affiliations:** ^1^ Department of Physics and Academy for Advanced Interdisciplinary Studies, Southern University of Science and Technology, Shenzhen, China; ^2^ 21C Innovation Laboratory, Contemporary Amperex Technology Ltd. (CATL), Ningde, China; ^3^ School of Materials Science and Engineering, Dongguan University of Technology, Dongguan, China; ^4^ Guangdong-Hong Kong-Macao Joint Laboratory for Neutron Scattering Science and Technology, Dongguan, China

**Keywords:** interfacial compatibility, thermal stability, solid-state electrolyte, halide, rock-salt, anti-perovskite, solid-state battery

## Abstract

All-solid-state lithium batteries (ASSLBs) have attracted much attention owing to their high safety and energy density compared to conventional organic electrolytes. However, the interfaces between solid-state electrolytes and electrodes retain some knotty problems regarding compatibility. Among the various SSEs investigated in recent years, halide SSEs exhibit relatively good interfacial compatibility. The temperature-dependent interfacial compatibility of halide SSEs in solid-state batteries is investigated by thermal analysis using simultaneous thermogravimetry and differential scanning calorimetry (TG–DSC) and X-ray diffraction (XRD). Halide SSEs, including rock-salt-type Li_3_InCl_6_ and anti-perovskite-type Li_2_OHCl, show good thermal stability with oxides LiCoO_2_, LiMn_2_O_4_, and Li_4_Ti_5_O_12_ up to 320 °C. Moreover, anti-perovskite-type Li_2_OHCl shows a chemical reactivity with other battery materials (eg., LiFePO_4_, LiNi_0.8_Co_0.1_Mn_0.1_O_2_, Si-C, and Li_1.3_Al_0.3_Ti_1.7_(PO_4_)_3_) at 320°C, which reaches the melting point of Li_2_OHCl. It indicated that Li_2_OHCl has relatively high chemical reactivity after melting. In contrast, rock-salt-type Li_3_InCl_6_ shows higher stability and interfacial compatibility. This work delivers insights into the selection of suitable battery materials with good compatibility for ASSLBs.

## 1 Introduction

As energy storage equipment, Li-ion batteries were widely applied in portable electronic devices and electric vehicles after decades of rapid development. However, commercial lithium-ion batteries exhibit obvious disadvantages due to the flammability and leakage of organic liquid electrolytes, which might lead to serious safety problems (Li et al., 2018). The safety of the battery could be enhanced by designing an all-solid-state lithium battery in which the liquid electrolyte is replaced with solid-state electrolytes (Wang et al., 2012; Manthiram et al., 2017). As the most critical component of an all-solid-state lithium battery, solid-state electrolytes (SSEs) require not only high ionic conductivity and wide electrochemical window but also good interfacial compatibility toward other battery materials to form stable interfaces in all-solid-state lithium batteries (ASSLBs) (Winter 2009). Nevertheless, building a stable interface remains a huge challenge; the exploitation of simple and effective instruments to study the compatibility of electrodes and electrolytes will facilitate the construction of stable interfaces. Recently, Ceder et al. proposed a methodology that combines density functional theory calculations and simple experimental techniques to study the interfacial compatibility between numerous electrolytes and electrodes and screened out more than 20 different electrode/electrolyte pairs with fine compatibility for Na solid-state batteries (Tian et al., 2017).

Over the past few decades, numerous SSEs have been exploited, which can be mainly classified into polymers, oxides, sulfides, and halides (Gao et al., 2018). Among them, halide SSEs are promising candidates for large-scale construction of ASSLBs due to their high ionic conductivity, relatively good interfacial compatibility, and easy preparation with a mechanical ball-milling approach and low-temperature sintering (Li et al., 2020). Rock-salt type Li_3_MCl_6_ (M = In, Y, Sc) and anti-perovskite type Li_3-*x*_OH_*x*_X (X = Cl, Br) are two typical halide SSEs. From the point of view of interface compatibility, there is a great deal of difference actually between them. For example, Li_3_YCl_6_ showed good electrochemical oxidation stability. The protected LiCoO_2_ using Li_3_YCl_6_ demonstrated a high initial Coulombic efficiency of 94.8%, in sharp contrast to that of 84.0% using Li_3_PS_4_ (Asano et al., 2018). However, Li_3_MCl_6_ displayed poor electrochemical reduction stability, impeding their application to lithium metal anode. Riegger et al. (2021) reported the instability of Li_3_InCl_6_ (Li_3_YCl_6_) with lithium metal and formed a passivation layer with high interfacial resistance by *in situ* X-ray spectroscopy and impedance spectrum. In contrast, Li_2_OHX (X = Cl, Br) showed good electrochemical reduction stability and had good stability against lithium metal anode (Hood et al., 2016). Although there are quite a few studies on these two classes of materials, the interfacial issues are not comprehensively and systematically researched up till now.

ASSLBs often require high densification to achieve high energy density and good interfacial contact with large contact areas through external pressure and heating treatment. However, this process usually accelerates the interdiffusion of elements at the interface, leading to the decomposition reaction. In this work, the temperature-dependent interfacial compatibility of halide SSEs (Li_3_InCl_6_ (LIC), Li_2_OHCl (LOHC)) with cathode (LiCoO_2_ (LCO), LiFePO_4_ (LFP), LiMn_2_O_4_ (LMO), Li-rich, LiNi_0.8_Co_0.1_Mn_0.1_O_2_ (LNMO), LiNi_0.5_Mn_1.5_O_4_ (LNMO)), anode (graphite, Si-C, Li_4_Ti_5_O_12_ (LTO), and SSEs (Li_1.3_Al_0.3_Ti_1.7_(PO_4_)_3_ (LATP), Li_10_GeP_2_S_12_ (LGPS)) were investigated by thermal analysis using simultaneous thermogravimetry and differential scanning calorimetry (TG–DSC) and X-ray diffraction (XRD). The results revealed that Li_2_OHCl has high chemical reactivity after melting, while the interfacial compatibility of Li_3_InCl_6_ is relatively good. This work provided insights into the selection of suitable battery materials with good compatibility for ASSLBs.

## 2 Experiment

### 2.1 Material preparation

LOHC powders were prepared using a solid-phase reaction. (Deng et al., 2020). The molar ratio was 1.05:1 for LiOH (99 wt%, Aladdin) and LiCl (99 wt%, Aladdin). First, LiOH (99 wt%, Aladdin) and LiCl (99 wt%, Aladdin) were weighed with the molar ratio of 1.05:1, ground, and mixed in a mortar. The mixture was then placed in a nickel crucible, heated to 400°C, held for 4 h, and cooled naturally. Finally, the product was ground into fine powder. Other battery materials, including LCO, LFP, LMO, Li-rich, NCM811, and LNMO cathode materials; graphite, Si-C, and LTO anode materials; LIC, LATP, and LGPS SSEs are all commercially available. Every battery material was mixed with either LIC or LOHC, respectively, in a mass ratio of 1:1. Sintering of the mixing powder was carried out in a nickel crucible at 170 and 320°C, respectively. The dwell time at each temperature was 4 hours, the heating rate was 5 K/min, and the cooling mode was natural cooling. After heating treatment, the sintered samples were ground into powder in a mortar for subsequent characterization.

### 2.2 Characterization

The crystallinity of samples was measured using PANalytical Diffraction System with Cu Kα radiation. Powders were protected from the moisture in the air using polyimide film. Phase analysis was performed using the Powder Diffraction Files (PDF) database (PDF, reference numbers are listed in Supplementary Table S1). A simultaneous thermal analyzer was carried out using NETZSCH STA 449F3 in an N_2_ atmosphere between room temperature and 350°C at a heating and cooling rate of 5 K/min.

## 3 Results and discussion

**FIGURE 1:**
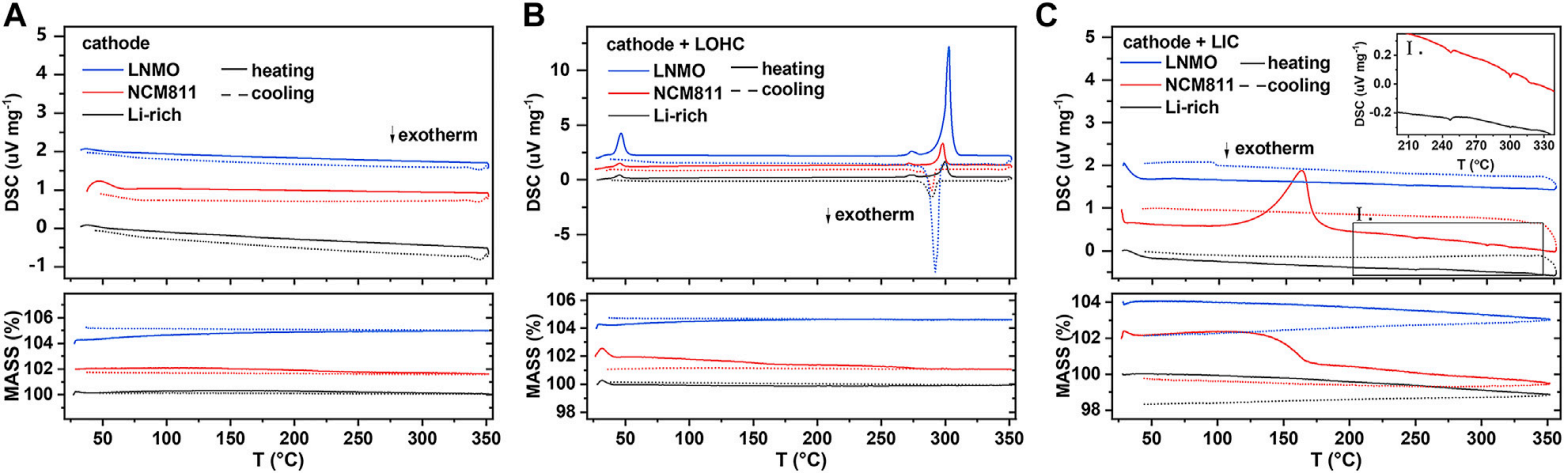
DSC and TG results of the cathode **(A)**, cathode/LOHC mixtures **(B)**, and cathode/LIC mixtures **(C)**. Inset Ⅰ is a magnification of the marked areas. DSC signal offset: 1 unit (for NCM811) and 2 units (for LNMO). TG signal offset: 2 units (for NCM811) and 4 units (for LNMO).

**FIGURE 2:**
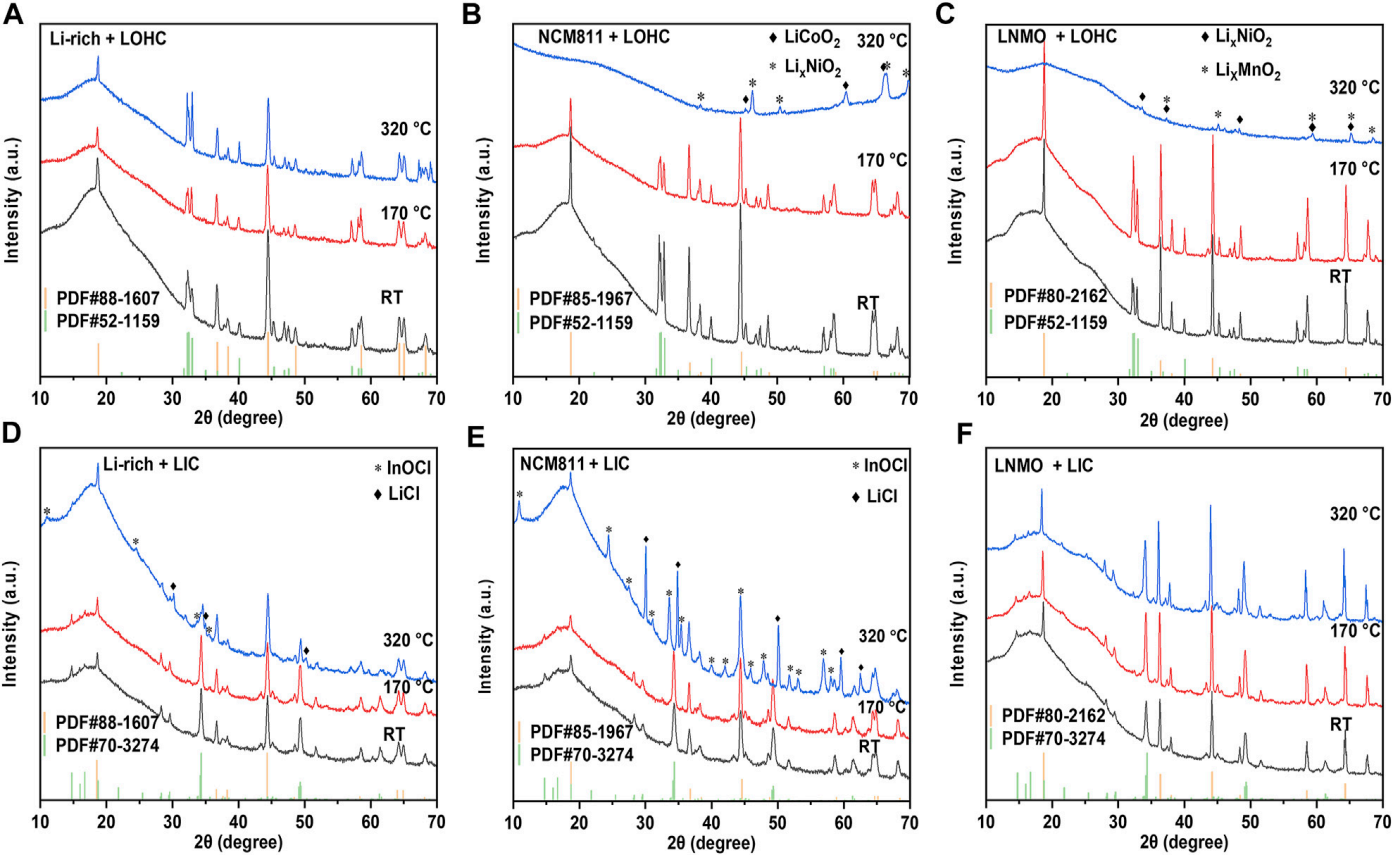
XRD patterns of Li-rich/LOHC **(A)**, NCM811/LOHC **(B)**, LNMO/LOHC **(C)**, Li-rich/LIC **(D)**, NCM811/LIC **(E)**, and LNMO/LIC **(F)** mixtures that were sintered at 170 and 320°C and mixed powder at room temperature. Supplementary Table S1 lists the consulted reference patterns.

As shown in Figure 1A and Supplementary Figure S1A, all TG–DSC curves of studied cathodes, including LNMO, NCM811, Li-rich, LMO, LCO, and LFP, show no obvious peak, indicating good thermal stability when below 350°C. Supplementary Figure S2 shows the TG–DSC curves of LOHC and LIC. The endothermic peak around 45°C during the heating process of LOHC indicates the phase transition from orthorhombic to cubic anti-perovskite phase, and the subsequent pair of endothermic and exothermic peaks corresponds to the melting and solidification process of LOHC. Although Figure 1B shows no other significant peaks, the XRD patterns shown in Figure 2 and Supplementary Figure S3 demonstrate obvious chemical reactions of NCM811/LNMO/LFP + LOHC mixtures after heating treatment at 320°C. NCM811 + LOHC mixtures indicate LiCoO_2_ and Li_*x*_NiO_2_ reflections, LNMO + LOHC mixtures indicate LiMnO_2_ and Li_*x*_NiO_2_ reflections, and LFP + LOHC mixtures indicate LiCl reflections. The overlap of reaction endothermic and melting endothermic is the result of a chemical reaction of these mixtures after LOHC melting. It is also confirmed that the XRD patterns of these mixtures after 170°C heating treatment exhibit no obvious impurity peak, indicating that they are thermally stable under relatively low temperatures. It is because molten LOHC has free hydroxide radicals and Cl^−^, presenting higher reactivity than the solid phase. By contrast, Li-rich, LCO, and LMO cathodes have higher compatibility with molten LOHC.

Compared to LOHC, LIC has a high melting temperature and good compatibility with such cathode materials as LNMO, LCO, LFP, and LMO. Both TG–DSC curves and XRD patterns show no obvious chemical reaction of their mixtures with LIC after being annealed at 320 °C (shown in Figures 1 and 2 and Supplementary Figures S1 and S3). However, NCM811 and Li-rich cathodes show poor thermal stability with LIC. A significantly irreversible endothermic peak around 170°C was observed in the DSC curve of NCM811 + LIC mixtures, corresponding to the formation of plenty of impurities of InOCl and LiCl, as shown in Figure 2E. In comparison, the DSC curve of the Li-rich + LIC mixture show relatively weaker exothermic peaks at higher temperatures (shown in Figure 1C) and fewer impurities of InOCl and LiCl (shown in Figure 2D). It indicates poor interfacial compatibility of LIC with NCM811/Li-rich cathode materials.

**FIGURE 3:**
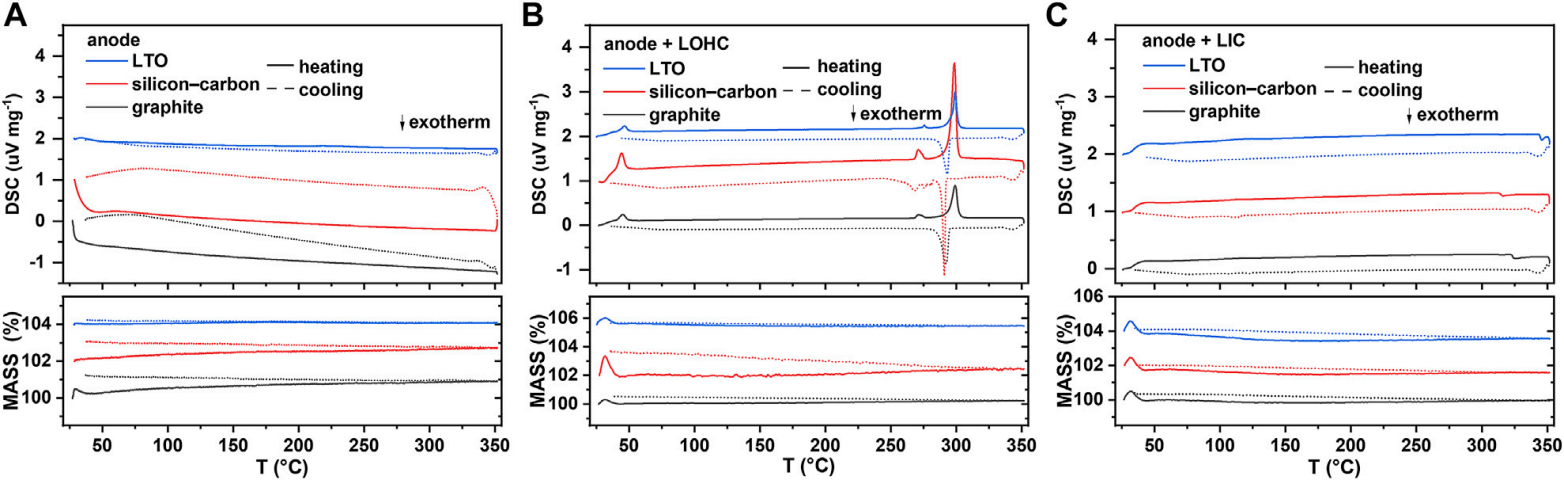
DSC and TG results of an anode **(A)**, anode/LOHC mixtures **(B)**, and anode/LIC mixtures **(C)**. DSC signal offset: 1 unit (for Si-C) and 2 units (for LTO). TG signal offset: 2 units (for Si-C) and 4 units (for LTO).

**FIGURE 4:**
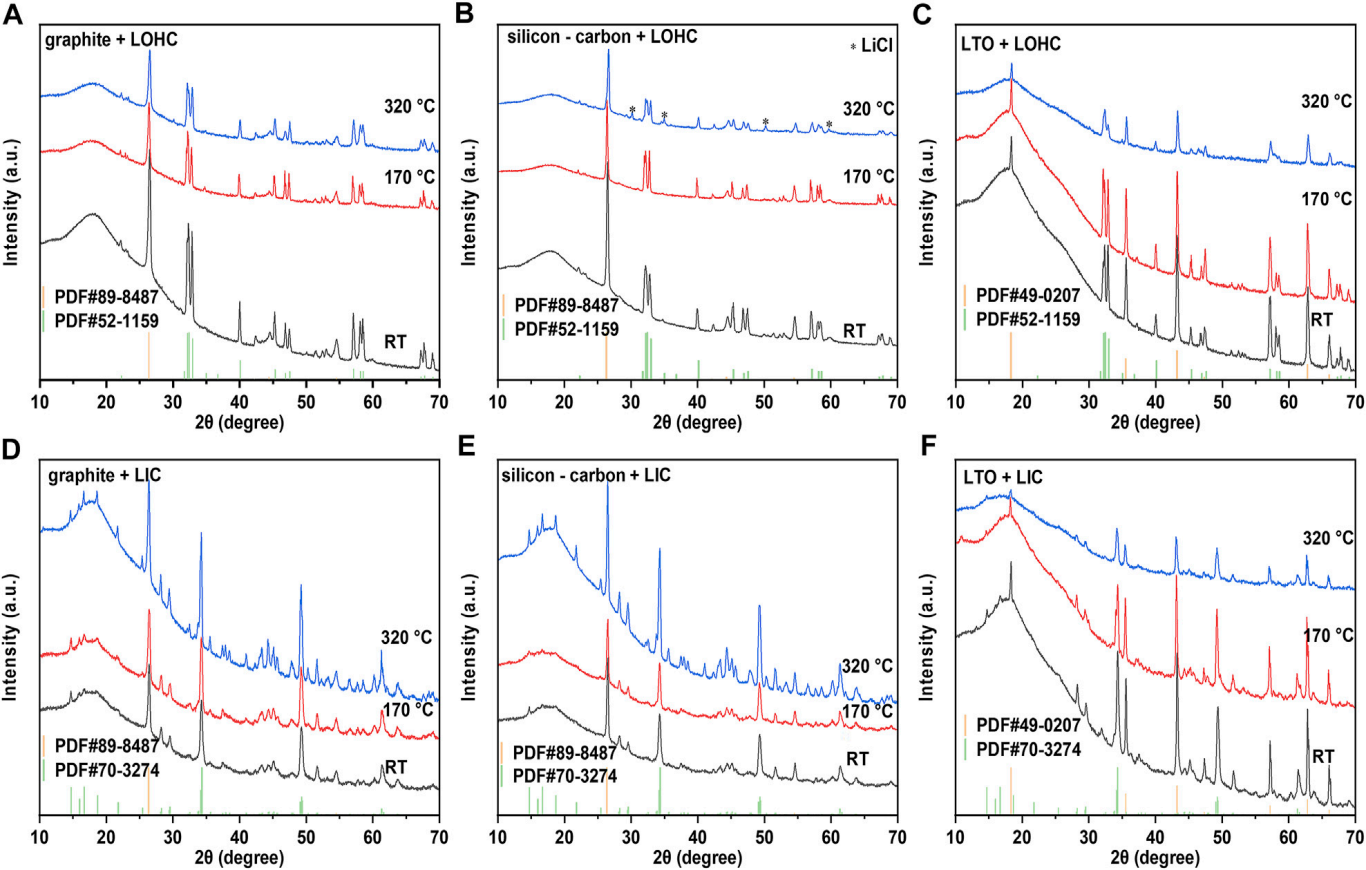
XRD patterns of graphite/LOHC **(A)**, Si-C/LOHC **(B)**, LTO/LOHC **(C)**, graphite/LIC **(D)**, Si-C/LIC **€**, and LTO/LIC **(F)** mixtures that were sintered at 170 and 320°C and mixed powder at room temperature. Supplementary Table S1 lists the consulted reference patterns.

Graphite, Si-C, and LTO, as three common anode materials, are chosen here to study the compatibility of the anode with halide SSEs. As shown in Figure 3A, graphite, Si-C, and Li_4_Ti_5_O_12_ exhibit good thermal stability, and the TG–DSC curves did not reveal any significant indications below 350°C. Similar to the TG–DSC curves of cathode + LOHC mixtures, Figure 3B shows no other thermal reaction of anode + LOHC mixtures, with the exception of the endothermic and exothermic peaks in LOHC. However, Figure 4B shows the formation of LiCl impurities after annealing at 320°C. It indicates that LOHC is thermally unstable with Si-C anode and that Si can be oxidized by the alkaline substance under heating to form silicate and releases hydrogen, the mass of which is ignorable and, thus, the mass change is not obvious in the TG curve (Figure 3B). LTO and graphite are naturally quite stable. Hence, graphite and LTO anode materials are comparatively stable with LOHC in expectation. As displayed in Figures 3 and 4, there is no obvious reaction indication in both TD–DSC and XRD patterns for LTO–LOHC and graphite–LOHC mixtures. Another positive finding is that both TG–DSC curves and XRD patterns of anode + LIC mixtures also show no apparent changes, indicating good thermal compatibility of these anode materials with LIC.

**FIGURE 5:**
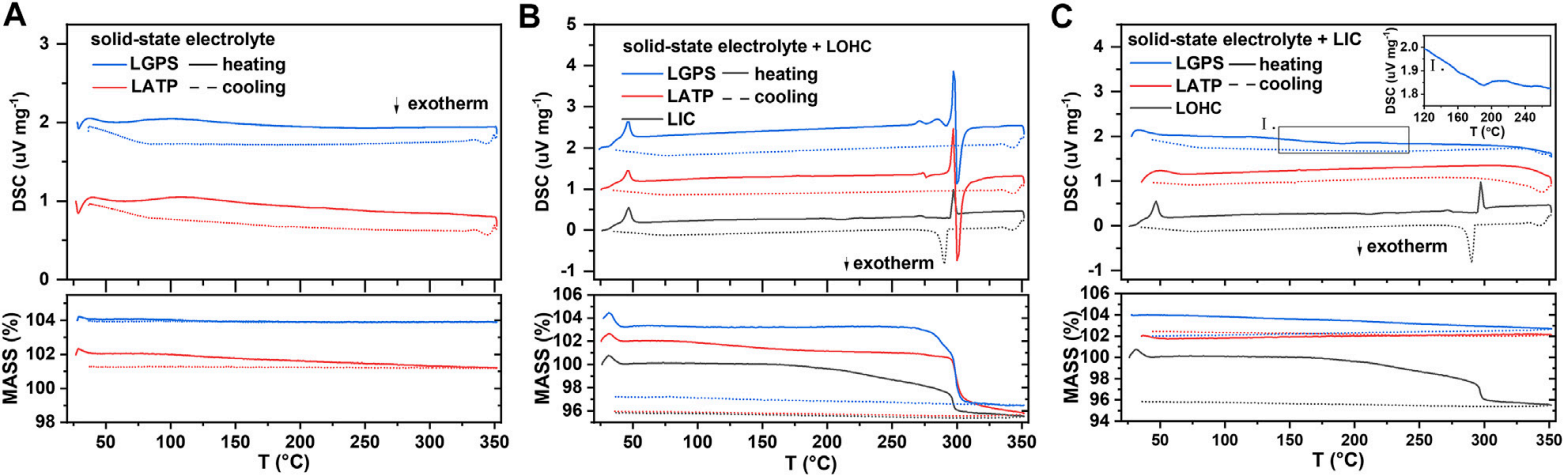
DSC and TG results of SSE **(A)**, SSE/LOHC mixtures **(B)**, and SSE/LIC mixtures **(C)**. DSC signal offset: 1 unit (for LATP) and 2 units (for LGPS). TG signal offset: 2 units (for LATP) and 4 units (for LGPS).

**FIGURE 6:**
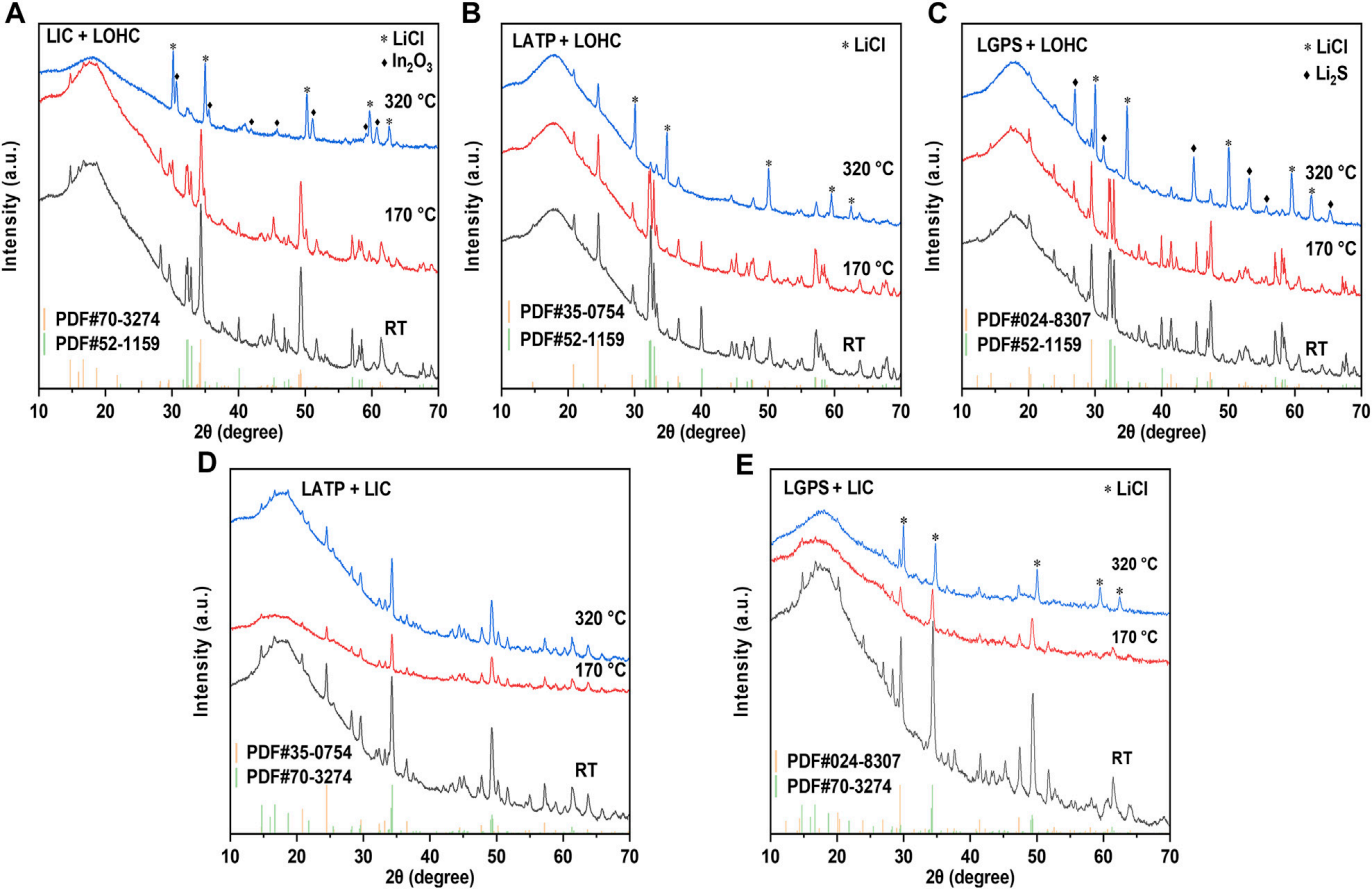
XRD patterns of LIC/LOHC **(A)**, LATP/LOHC **(B)**, LGPS/LOHC **(C)**, LATP/LIC **(D)**, and LGPS/LIC **(E)** mixtures that were sintered at 170 and 320°C and mixed powder at room temperature. Supplementary Table S1 lists the consulted reference patterns.

To address the interfacial issues, compounding various electrolytes has also been proposed to combine their respective advantages. (Zhao et al., 2016; Wang et al., 2022). Therefore, the interfacial compatibility between electrolytes also needs to be studied further. Figure 5 shows the TG–DSC results of the SSEs and the mixtures of SSEs with LOHC or LIC. The DSC curve in Figure 5A shows that LGPS and LATP are thermally stable at the measured temperature range, with no significant endo- or exo-thermic reaction and no evident mass change. The thermal characteristics of three SSE + LOHC mixtures are shown in Figure 5B after the initial phase transition of LOHC at around 45°C and the melting process of LOHC at 297°C, an exothermic reaction was observed at 299°C for LATP + LOHC and LGPS + LOHC mixtures. In addition, with regard to these three mixtures, this process shows a significant mass loss, which means a decomposition reaction occurs in the SSE + LOHC mixtures, accompanied by the release of H_2_O. The XRD patterns of the SSE + LOHC mixtures are shown in Figure 6. Corresponding to the thermal analysis results, for LATP + LOHC and LGPS + LOHC mixtures, the observed diffraction pattern of the sample sintered at 170°C is the same as that of unsintered mixed powder, and the peaks can be indexed with SSE, Li_2_OHCl, or Li_3_InCl_6_. After sintering at 320°C, LIC + LOHC mixtures form LiCl and In_2_O_3_ impurities. LATP + LOHC mixtures form LiCl impurity, while LGPS + LOHC mixtures form LiCl and Li_2_S impurities. As shown in Figure 5C, LGPS + LIC mixtures have an exothermic reaction at 190°C, without obvious mass change during the whole measured temperature range. LGPS + LIC mixtures form LiCl impurity. In contrast, LATP + LIC mixtures exhibit stable under 350°C without thermal reaction and XRD changes, as shown in Figure 5C and Figures 6D and E.

The halide electrolytes LOHC and LIC show comparable behavior during the co-sintering process in solid-state battery systems. All mixtures remained stable after sintering at 170°C. However, several mixtures reacted to form some impurity after sintering at 320°C. As shown in Table 1, NCM811 and LNMO show comparable behavior. After co-sintering with LOHC at 320°C, NCM811 and LNMO decompose into oxide cathode Li_*x*_NiO_2_, LiCoO_2,_ and LiMnO_2_. The phase transition process might be related to the delithiation and cation mixing process at a heating temperature of 320°C (Maleki KheimehHu et al., 2013; Noh et al., 2013; Sari and Li, 2019). LOHC decomposes into LiCl during the co-sintering process at 320°C with LFP cathode, Si-C anode, LIC, LGPS, and LATP SSE, accompanied by the release of H_2_O vapor. Decomposition products such as LiCl generally have low ionic conductivity, which results in a large interfacial resistance for Li^+^ conduction. The relatively high reactivity of LOHC at 320°C is mainly due to the presence of free hydroxide radicals and Cl^−^ in molten LOHC. Hence, compounds that are stable against alkaline compounds have a relatively good thermal compatibility with LOHC, such as Li-rich, LCO, and LMO cathode, graphite, and LTO anode. In contrast, LIC, as one type of metal halide SSE, has a higher melting temperature that exceeded the studied temperature range in this work. However, the relatively weak In−Cl bond also can be broken by the element with higher electronegativity, such as oxygen or hydroxide. As expected, LIC reacts with Li-rich and NCM811 cathode, LOHC and LGPS SSEs and forms impurities such as LiCl, InOCl, and In_2_O_3_.

**TABLE 1:** Battery materials with LOHC and battery materials with LIC sintered at 320°C, and the phases found in XRD. Red phases are formed after sintering.

Sample	XRD pattern after being sintered at 320°C	Newly generated phase
LCO/LOHC	LiCoO_2_ and Li_2_OHCl	
LCO/LIC	LiCoO_2_ and Li_3_InCl_6_	
LFP/LOHC	LiFePO_4_, Li_2_OHCl, and LiCl	LiCl
LFP/LIC	LiFePO_4_ and Li_3_InCl_6_	
LMO/LOHC	LiMn_2_O_4_ and Li_2_OHCl	
LMO/LIC	LiMn_2_O_4_ and Li_3_InCl_6_	
Li-rich/LOHC	Li-rich and Li_2_OHCl	
Li-rich/LIC	Li-rich, Li_3_InCl_6_, InOCl, and LiCl	InOCl and LiCl
NCM811/LOHC	LiCoO_2_ and Li_x_NiO_2_	LiCoO_2_ and Li_x_NiO_2_
NCM811/LIC	LiNi_0.8_Co_0.1_Mn_0.1_O_2_, InOCl, and LiCl	InOCl and LiCl
LNMO/LOHC	LiMnO_2_ and Li_x_NiO_2_	LiMnO_2_ and Li_x_NiO_2_
LNMO/LIC	LiNi_0.5_Mn_1.5_O_4_ and Li_3_InCl_6_	
Graphite/LOHC	Graphite and Li_2_OHCl	
Graphite/LIC	Graphite and Li_3_InCl_6_	
Si-C/LOHC	Si-C, Li_2_OHCl, and LiCl	LiCl
Silicon–carbon/LIC	Si-C and Li_3_InCl_6_	
LTO/LOHC	Li_4_Ti_5_O_12_ and Li_2_OHCl	
LTO/LIC	Li_4_Ti_5_O_12_ and Li_3_InCl_6_	
LATP/LOHC	Li_1.3_Al_0.3_Ti_1.7_(PO_4_)_3_ and LiCl	LiCl
LATP/LIC	Li_1.3_Al_0.3_Ti_1.7_(PO_4_)_3_ and Li_3_InCl_6_	
LGPS/LOHC	Li_10_GeP_2_S_12_, LiCl, and Li_2_S	LiCl and Li_2_S
LGPS/LIC	Li_10_GeP_2_S_12_, Li_3_InCl_6,_ and LiCl	LiCl
LIC/LOHC	Li_2_OHCl, LiCl, and In_2_O_3_	LiCl and In_2_O_3_

## 4 Conclusion

In summary, we explore the temperature-dependent interfacial compatibility of halide SSEs in solid-state batteries to find suitable candidates for designing ASSLBs. Our results show that both anti-perovskite LOHC and rock-salt type LIC halide SSEs are compatible with other battery materials, including some common cathode, anode, and SSE materials at a relatively low temperature, such as 170 °C. However, with increased temperature over 300°C, free hydroxide radicals in molten LOHC and weaker In−Cl bond have higher chemical reactivity than some other compounds. The reactions with LOHC are generally accompanied by the production of water vapor, corresponding to the mass loss in TG curves. On the contrary, the reactions with LIC generally generate In-based oxides such as InOCl and In_2_O_3_ without mass loss. In addition, LiCl is a usual impurity as a reaction product. However, it should be noted that these decomposition products are generally electronic insulators that can hinder the further interfacial reaction in ASSLBs. This work provides insight into the selection of suitable battery materials with good compatibility in ASSLBs, which is of great significance to future solid-state battery research.

## Data Availability

The original contributions presented in the study are included in the article/Supplementary Material; further inquiries can be directed to the corresponding authors.
