# Supplementary material for: Temperature-dependent compatibility study on halide solid-state electrolytes in solid-state batteries
Source: Front Chem. 2022 Aug 3;10:952875. doi: 10.3389/fchem.2022.952875 (PMC9382350; doi:10.3389/fchem.2022.952875)
Supplement: Supplementary file 1 [file DataSheet1.docx]

Supplementary Material

**Table S1.** Used reference patterns for qualitative phase analysis

| Phase | PDF number |
| --- | --- |
| LiCoO_2_ | PDF#50-0653 |
| LiFePO_4_ | PDF#83-2092 |
| LiMn_2_O_4_ | PDF#88-1749 |
| Li_0.51_Ni_1.16_O_2_ | PDF#70-3274 |
| Li_0.99_Ni_0.01_NiO_2_ | PDF#85-1967 |
| LiNi_0.5_Mn_1.5_O_4_ | PDF#80-2162 |
| Graphite | PDF#89-8487 |
| Li_4_Ti_5_O_12_ | PDF#49-0207 |
| Li_3_InCl_6_ | PDF#70-3274 |
| Li_2_OHCl | PDF#52-1159 |
| LiTi_2_(PO_4_)_3_ | PDF#35-0754 |
| Li_10_GeP_2_S_12_ | PDF#024-8307 |
| Li_0.06_NiO_2_ | PDF#49-0035 |
| InOCl | PDF#73-1560 |
| LiCl | PDF#04-0664 |
| LiOH | PDF#32-0564 |
| LiO | PDF#74-0115 |
| In_2_O_3_ | PDF#06-0416 |
| Li_3_PO_4_ | PDF#48-0956 |
| AlPO_4_ | PDF#45-0509 |
| Li_2_S | PDF#23-0369 |
| GeP_5_ | PDF#24-0455 |

**
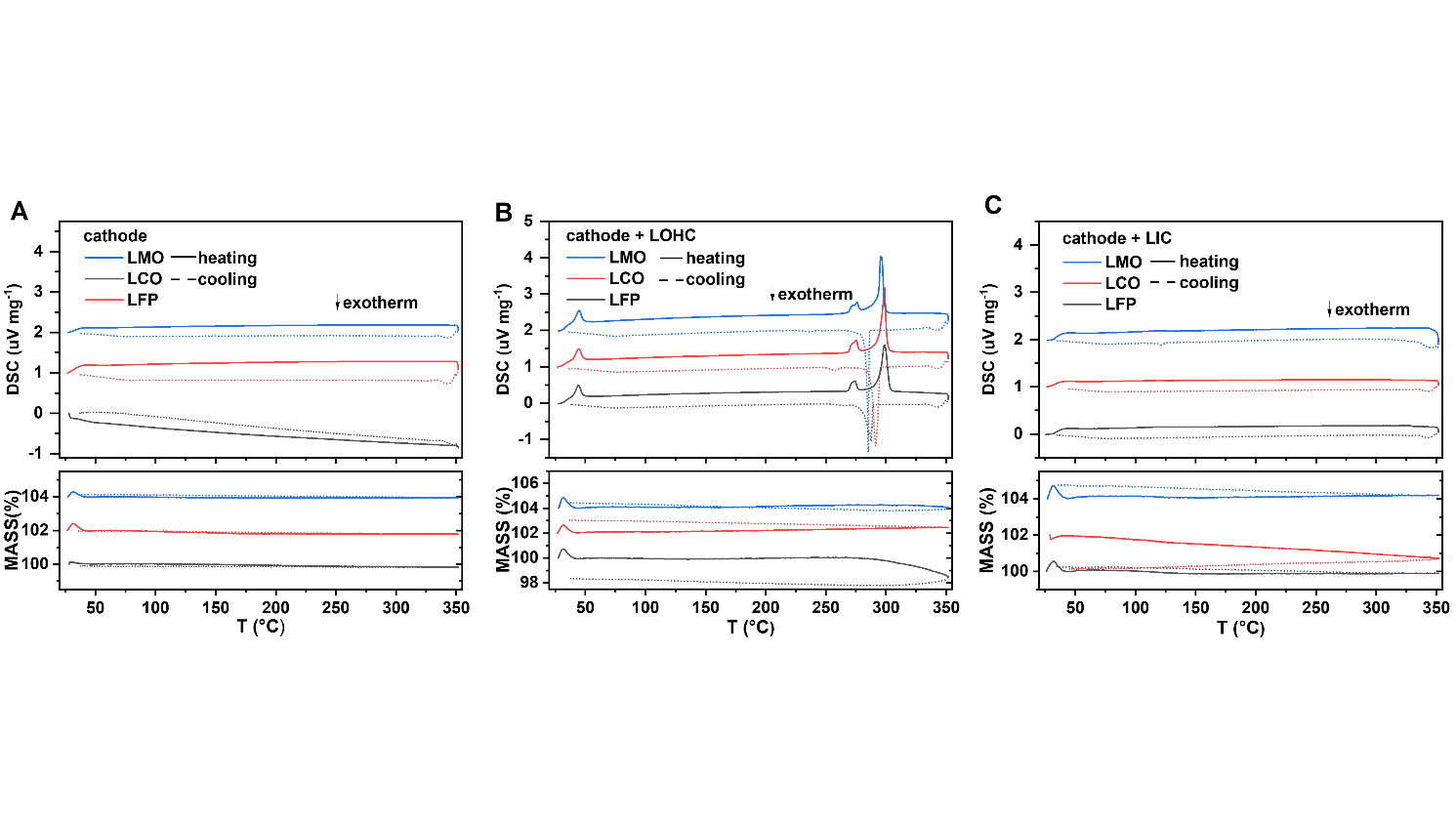
**. **FIGURE S1.** DSC and TG results of cathode (A), Cathode/LOHC mixtures (B) and Cathode/LIC mixtures (C). DSC signal offset: 1 unit (for LCO); 2 units (for LMO). TG signal offset: 2 units (for LCO) and 4 units (for LMO).


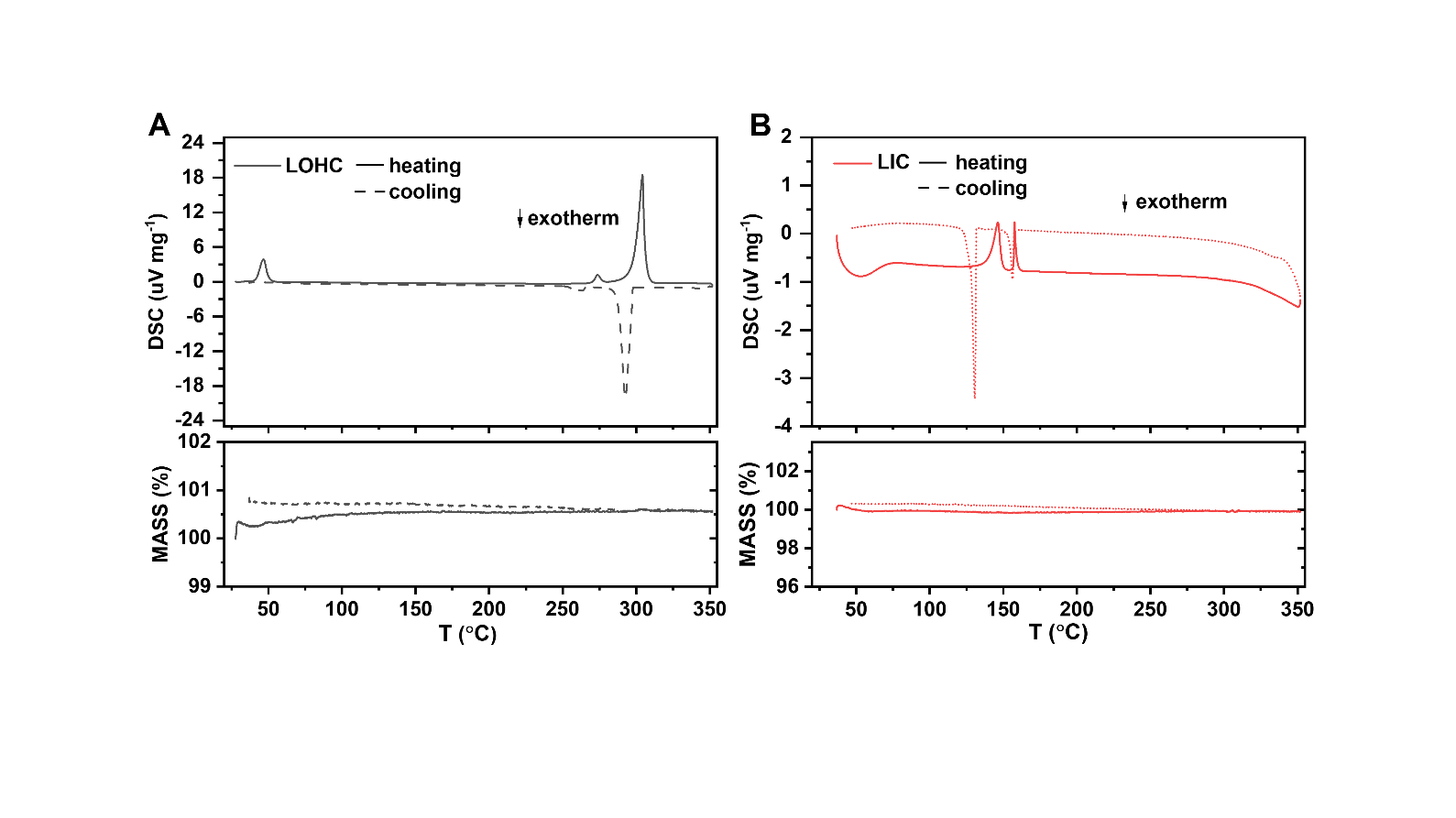


**FIGURE S2**. DSC and TG results of Li_2_OHCl (A), Li_3_InCl_6_ (B)

**
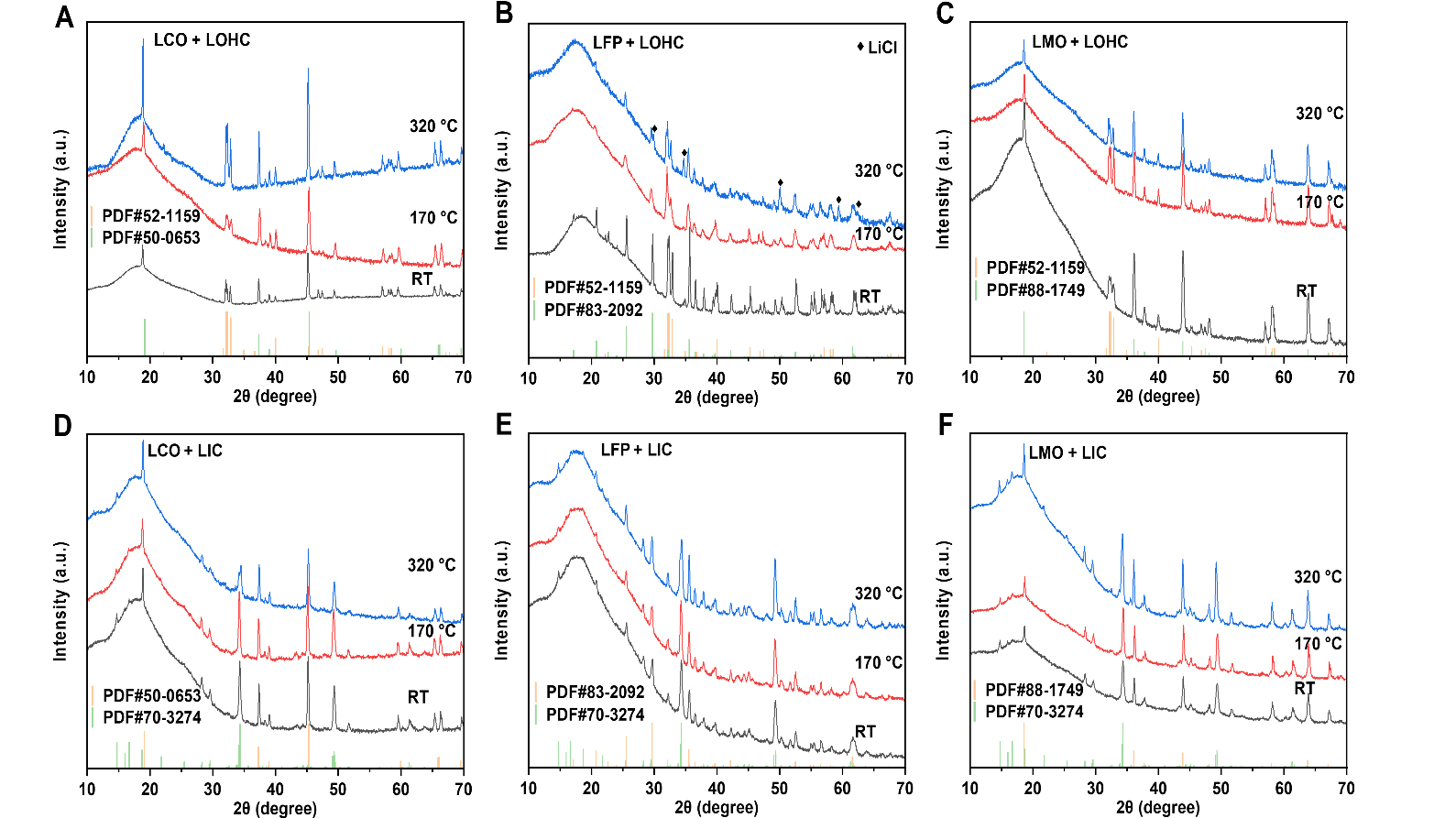
**

**FIGURE S3**. XRD patterns of LCO/LOHC (A), LFP/LOHC (B), LMO/LOHC (C), LCO/LIC (D), LFP/LIC (E), LMO/LIC (F) mixtures that were sintered at 170, 320 °C and mixed powder at room temperature. Table S1 lists the consulted reference patterns.
